# “You lose a day for every appointment”: A qualitative study of how rural versus urban residence shapes cancer care experiences in Northeast Scotland

**DOI:** 10.1007/s00520-026-10836-2

**Published:** 2026-06-04

**Authors:** Romi Carriere, Rosalind Adam, Leslie Samuel, Peter Murchie

**Affiliations:** 1 Myeloma UK, Edinburgh, Scotland, UK; 2 Academic Primary Care Research Group, Institute of Applied Health Sciences, University of Aberdeen, Aberdeen, Scotland, UK; 3 Department of Oncology, Aberdeen Royal Infirmary, NHS Grampian, Aberdeen, Scotland, UK

**Keywords:** Rural health, Cancer care, Qualitative research, Scotland, Patient experience, Travel burden

## Abstract

**Purpose:**

Quantitative studies show that rural-dwelling cancer patients experience poorer survival than urban residents, but the mechanisms remain unclear. This study explored how rural versus urban residence shaped cancer care experiences among patients interviewed after primary treatment in Northeast Scotland, including treatment access, follow-up, recovery, and ongoing engagement with services.

**Methods:**

Semi-structured interviews were conducted with adults diagnosed within the previous 6–12 months, post-primary treatment, and attending oncology follow-up at Aberdeen Royal Infirmary. Topic guides were informed by a socioecological model of rural cancer disadvantage. Interviews were audio-recorded, transcribed verbatim, anonymised, and analysed using the Framework Method. Sampling was purposive for geography, age, and sex. Ethical approval was granted by the North of Scotland Research Ethics Committee (REC 19/NS/0032).

**Results:**

Twenty participants (mean age 67; 13 men, 7 women; 9 rural, 11 urban) described experiences of colorectal (*n* = 12), prostate (*n* = 3), and other cancers (*n* = 5). Four themes were identified: (1) impact of distance and travel time—travel amplified fatigue, costs, and uncertainty; (2) access, trust, and communication—variable GP access but high trust in the cancer centre; (3) physical and social infrastructure—benefits of improved roads, direct bus routes, and third-sector support; (4) pros and cons of rural life—rural lifestyle benefits often offset travel burdens. Men tended to rely mainly on partners, while women reported broader networks. Cancer type and age intersected with geography to shape burdens such as continence-related travel constraints after prostate surgery.

**Conclusions:**

Geography influenced the burden of accessing rather than the quality of care. Person-centred scheduling, local delivery of peripheral care, transport-aware planning, and collaboration with third-sector providers could mitigate inequities.

**Supplementary Information:**

The online version contains supplementary material available at 10.1007/s00520-026-10836-2.

## Introduction

Population-based studies consistently demonstrate that people living in rural areas experience poorer cancer survival than their urban counterparts [1]. These challenges are not unique to Scotland. International rural-health literature, including a Canadian qualitative meta-synthesis, has identified distance, transport availability, and care coordination as recurrent barriers to accessing health care in rural and remote settings [2]. Cancer-specific studies from rural Australia similarly describe the practical and psychosocial burden of travelling for care, being away from specialist centres, and negotiating support needs across families and services [3, 4]. This wider evidence suggests that rural cancer disadvantage is not simply a matter of geography but reflects interactions between patients, services, communities, and policy contexts. Our previous systematic review developed a socioecological model suggesting that this disparity reflects interacting influences at individual, institutional, community, and policy levels rather than a single cause [1]. These influences may be particularly important after diagnosis, when patients must repeatedly engage with specialist services for treatment, monitoring, follow-up, and management of ongoing effects. For rural patients, this phase may involve substantial travel, time, cost, and symptom-related burdens, yet quantitative studies cannot fully explain how geography, service design, and lived experience interact to shape care.

This qualitative study builds on that framework by examining how these factors operate in practice within Northeast Scotland, where urban, rural, remote rural, and island communities are served by a single regional cancer centre. Recent quantitative analyses confirm that travel-related challenges persist and have highlighted the cumulative “travel toxicity” experienced by rural patients [5, 6]. While recent qualitative and mixed-methods work has explored cancer experiences in rural and island settings, few studies have examined these alongside urban comparators [7–10]. By integrating both perspectives within a single regional system, the present study illuminates how structural and experiential differences shape patients’ engagement with cancer care after diagnosis, including treatment, follow-up, and recovery.

Scottish data suggest that rural patients may be particularly vulnerable in the early post-diagnosis period with poorer outcomes despite comparable diagnostic timelines [5, 11]. The present study explores how geography and travel demands shape patients’ experiences and decision-making after diagnosis and primary treatment, to inform equitable service design in regions characterised by long travel distances, island communities, and ageing populations. We therefore undertook a qualitative interview study with individuals from Northeast Scotland living in rural and urban settings who had completed primary treatment and were attending specialist oncology follow-up at the regional cancer centre. We aimed to explore how residential geography shaped experiences after diagnosis and primary treatment, including treatment access, follow-up, recovery, and ongoing engagement with services.

## Methods

### Study setting

Northeast Scotland includes urban, rural, remote rural, and island communities, served by a single regional cancer centre at Aberdeen Royal Infirmary (ARI), and reflects the full range of settings described in the Scottish Government rural–urban classification [12].

### Design and participants

We conducted a qualitative interview study with adults (≥ 18 years) diagnosed within the previous 6–12 months, who had completed primary treatment and were attending oncology follow-up at Aberdeen Royal Infirmary. Eligibility was based on time since diagnosis rather than a fixed interval from completion of treatment to interview. The exact interval between completion of primary treatment and interview was not recorded consistently; therefore, the study is best understood as examining early experiences after diagnosis and primary treatment, rather than longer-term survivorship or late effects. Any stage and cancer type were eligible; participants were English-speaking and had capacity to be interviewed. We used purposive recruitment to achieve variation in residential geography, sex, and age. Residential geography was assigned from participant postcode using the Scottish Government Urban Rural Classification two-fold measure [12]. This is an area-based classification derived from settlement size: settlements with populations of fewer than 3000 people are classified as rural, while all other areas are classified as urban. It therefore reflects the settlement context of the participant’s postcode rather than individual travel distance to Aberdeen Royal Infirmary. Potential participants were identified prospectively during oncology follow-up clinics. Members of the oncology team assessed eligibility during routine appointments and asked suitable patients whether they would be willing to speak with the researcher about the study. Patients who expressed interest were introduced to RC in clinic, where she provided verbal and written information. RC subsequently contacted interested patients to confirm willingness to participate, obtain consent, and arrange the interview. Participants supplied sex, date of birth, and postcode to derive Scottish Government urban/rural status and SIMD deprivation quintile [12, 13].

### Topic guide and data collection

A semi-structured topic guide was developed from the socioecological model described by Carriere et al. [1] and reviewed by the study team before use (Supplementary Material 1) [1]. The interviews were designed to explore rural and urban cancer care experiences broadly, rather than post-treatment survivorship alone. Sections explored individual factors (background, help-seeking, and experience of cancer), institutions (access, travel, and scheduling), community factors (sense of community and talking about cancer), and policy/service organisation (views on local service configuration). Interviews began with an open narrative of each participant’s cancer journey. Because participants were recruited after primary treatment and while attending oncology follow-up, their accounts included reflections on diagnosis and treatment as well as follow-up, recovery, and ongoing engagement with services.

Interviews were conducted by RC (female), audio-recorded, transcribed verbatim, anonymised, and accompanied by an appended demographics sheet (age, sex, SIMD quintile, two-fold urban/rural, cancer type). Participants chose face-to-face (University of Aberdeen, Centre of Academic Primary Care) or telephone. Data collection continued until the team judged that concepts were sufficiently explored across key strata.

### Analysis

We used the Framework Method [14]. RC read all transcripts, developed a provisional coding framework, and constructed a matrix (participants as rows; topics/themes as columns) populated with summarised data and illustrative quotes. RA and PM read a sample of transcripts, reviewed the developing framework and matrix, and agreed refinements. RC then applied the agreed framework to all transcripts and charted data into an Excel matrix to support comparison within and across cases. Divergences were discussed in team meetings. The core team comprised academic primary care clinicians (RA, PM) and a qualitative researcher (RC). Team members brought clinical and service-design experience of the local context. We maintained an audit trail (topic guide iterations, coding decisions, matrix versions) and used investigator triangulation to enhance credibility.

## Results

### Participants

Twenty of 28 consenting individuals completed interviews (five did not respond to further contact; three subsequently declined) (Table 1). Mean age was 67 years (range 42–81); 13 men and seven women; nine rural, eleven urban (per Scottish two-fold classification). Cancers included colorectal (*n* = 12), prostate (*n* = 3), and one each of anal, breast, cervical, GIST, and testicular. Most interviews were by telephone (*n* = 17); three were in person. Interviews lasted 15–37 min and were conducted between July 2019 and January 2020.

**Table 1:** Characteristics of sample

Variable	*N*, total (*N*%)
	*N* = 20
Mean age (SD; range)	67.2 (10.0; 42–81)
Age < 60 years	5 (25.0)
Age 60–69 years	6 (30.0)
Age ≥ 70 years	9 (45.0)
Gender	
Male	13 (65.0)
Female	7 (35.0)
Deprivations (SIMD quintile)	
SIMD Q1 (most)	-
SIMD Q2	-
SIMD Q3	5 (25.0)
SIMD Q4	8 (40.0)
SIMD Q5 (least)	7 (35.0)
Urban and rural patients (postcode UR2)	
Urban	11 (55.0)
Rural	9 (45.0)
Cancer type	
Anal	1 (5.0)
Colorectal	12 (60.0)
Breast	1 (5.0)
Cervical	1 (5.0)
Gastrointestinal stromal tumour (GIST)	1 (5.0)
Prostate	3 (15.0)
Testicular	1 (5.0)

### Emergent themes

Although participants were recruited after primary treatment and while attending oncology follow-up, their accounts often moved across diagnosis, treatment, recovery, and follow-up. We therefore present findings as early post-diagnosis and post-primary-treatment cancer care experiences, with particular attention to how geography shaped treatment access, recovery, follow-up, and ongoing engagement with services. Four inter-related themes were identified: distance and travel time; access, trust, and communication; physical and social infrastructure; and the perceived pros and cons of rural life. Across themes, the main rural–urban contrast was not that rural participants perceived poorer specialist care but that they described greater logistical, financial, and symptom-related work in accessing care. Rural participants also tended to normalise travel burden as part of rural life, whereas urban participants more often viewed distance from Aberdeen as a clear disadvantage for people living further from the cancer centre.

### Impact of distance and travel time

Participants frequently described the journey to Aberdeen Royal Infirmary (ARI) as one of the most demanding aspects of engaging with cancer care after diagnosis. For many, distance and travel time amplified the physical and emotional strain of treatment, recovery, and follow-up. A rural participant who lived more than 50 miles from Aberdeen recalled:

They sent you home with a bowl. I had to pee in that… it was really that bad. (M, 52, rural)

Ongoing side effects such as incontinence, fatigue, and nausea made long trips particularly challenging, leading some to limit social contact or postpone follow-up appointments. The same man reflected,

I can’t go to see friends because I’d need to take a bag of nappies under my arm.

For others, long travel days were simply exhausting:

You lose a day for every appointment. (F, 70, rural)

Unpredictable scheduling and long waiting times at the hospital added to the challenge. One patient described arriving for chemotherapy at 8 a.m. but not receiving treatment until late afternoon, forcing an unplanned overnight stay. Another, who needed morning appointments due to fatigue, explained:

They gave me an afternoon slot and I just couldn’t manage it, so I missed that one. (F, 59, urban)

Travel difficulties sometimes delayed help-seeking. A woman recovering from chemotherapy reflected that she might have sought advice sooner if she had lived closer:

I might have popped into the hospital and said, ‘Am I meant to feel this ill?’ Instead, I just sat at home. (F, 70, rural)

Financial implications were also evident. Fuel costs mounted quickly, particularly for those who had reduced work hours or were on sick leave. One man commented,

It’s 51 miles each way, and I was off work eight weeks. The petrol alone was a stretch. (M, 52, rural)

Even some urban participants described transport barriers when they lacked a car or reliable late-night public transport:

They could only give the tablets at A&E, but I had no way to get there, so I just waited until morning. (M, 43, urban)

Across interviews, participants accepted the necessity of specialist care at ARI but highlighted the effort required to access it. Rural patients tended to normalise the burden as “part of country life”, while urban participants often perceived rurality as a disadvantage. Taken together, these accounts suggest that travel distance did not determine whether care was received, but strongly shaped how it was experienced, including fatigue, costs, self-management decisions, and isolation from services.

### Access, trust, and communication

Access to care was described as variable, shaped more by appointment systems, continuity, and communication than by geography itself. Most participants expressed strong confidence in the specialist cancer centre, but many found it harder to engage with local primary care. One rural participant explained:

It’s almost impossible to get an appointment with my GP, so I just went into Aberdeen every time I needed bloods. It was less hassle. (M, 68, rural)

Several participants, both urban and rural, echoed this frustration, linking appointment barriers to a loss of continuity and trust. A woman from the city commented,

We never get the same doctor. There’s no relationship anymore, and it’s so difficult to get an appointment. (F, 67, urban)

He’s a busy man… I didn’t want to waste his time with something minor like an irritated backside. (M, 80, rural)

Conversely, where a continuing relationship existed, patients described quick and reassuring responses.

I’ve got a lovely lady GP who didn’t waste any time. I was lucky — she just got things done. (F, 68, urban)

Administrative glitches or problems at the primary–secondary care interface created anxiety and a sense of neglect. One man discovered that he had been “lost in the system”, and a recurrence was only diagnosed once he chased up missed follow-up tests. Another participant (M, 80, rural, anal cancer) described how his diagnosis was delayed after surgery when results were slow to be shared between his local hospital and Aberdeen. For others, even minor administrative lapses had an emotional impact.

The receptionist didn’t want to give me an appointment, even though they should have been expecting me. She said the messages take days to come through. (M, 68, rural)

For an island and more remote participants, specialist referral often restored confidence after uncertainty in local care. One man described a prolonged delay before diagnosis:

It took 18 months before they did a PSA test — my neighbour actually told me to ask for one. Once Aberdeen got involved, they took an interest and things moved quickly. (M, 76, rural [island])

Across settings, clear and responsive communication was described as the foundation of trust. When contact was swift and information flowed easily, patients felt valued and safe. One woman recalled:

The GP saw me within an hour, sent me to hospital the same day, and I was diagnosed within two hours. They were amazing. (F, 55, rural)

Overall, participants contrasted the professionalism and efficiency of specialist oncology care with the often-fragmented nature of general practice access. Geography itself did not determine perceived quality; rather, trust arose from continuity, communication, and responsiveness, which patients viewed as important features of good care wherever they lived.

### Physical and social infrastructure

Participants linked their experiences to infrastructure supporting access, including roads, transport, local facilities, and community or charitable support. Improvements to regional transport networks, particularly the Aberdeen Western Peripheral Route (AWPR), were repeatedly cited as beneficial.

Before they built the new road, it could take over an hour. We can do it in forty minutes now. (M, 64, rural)

The bypass has made a big difference — getting in and out of Aberdeen is far less stressful. (F, 59, rural)

Public transport options also determined access, especially for those unable or unwilling to drive long distances. Some described direct bus links to Aberdeen as crucial for maintaining independence and reported differing experiences:

I just walk up to the square and the bus takes me straight to the hospital door — it’s perfect. (M, 68, rural)

For others, however, travel still involved multiple connections or unpredictable timetables:

To get there I need two buses, and that makes it hard to be on time. (F, 70, urban)

Local health service infrastructure, such as being able to have blood tests at GP surgeries, was valued for reducing unnecessary journeys. Rural participants, in particular, noted how this availability allowed them to engage with hospital-based care more easily.

Getting the bloods done at my surgery in the morning before chemo made all the difference. (M, 64, rural)

Several participants highlighted the essential role of third-sector organisations in bridging gaps left by geography. CLAN (Cancer Link Aberdeen and North) was praised for offering nearby accommodation and emotional support during treatment:

I stayed at CLAN while I was on radiotherapy — it was so convenient, and if you didn’t feel like walking, they’d get you there. (M, 80, rural)

Friends of ANCHOR, a charity providing in-hospital support, were also mentioned with gratitude:

They really helped — they made a massive difference in the chemotherapy ward. (M, 65, rural)

Social and emotional support extended beyond formal organisations. Participants described strong networks of spouses, family, and neighbours who played practical and emotional roles during treatment.

My husband came to every appointment. I was too nervous to ask questions, but he always did. (F, 58, urban)

A neighbour who’d had prostate cancer told me to ask for a PSA test — that’s what got me diagnosed. (M, 76, rural (island))

Together, these accounts demonstrate that infrastructure, physical, organisational, and social, was central to how cancer care was experienced. Well-functioning transport routes, local service capacity, and community or charitable support mitigated some rural disadvantages, enabling patients to engage with care on more equal terms despite the geographical challenges they faced.

### Pros and cons of rural life

Participants expressed a nuanced understanding of how rural living shaped their experiences of cancer and healthcare. For most, rural life was deeply tied to identity and wellbeing. Even when travel burden was acknowledged, it was often balanced by an appreciation for space, tranquillity, and community.

Life out here couldn’t be better — cleaner air, peace, no traffic. I’ve been farming all my days, and I wouldn’t trade it. (M, 81, rural)

We live in the country, the kids have horses — it’s just a simpler way of life. (M, 64, rural)

Several participants described rural living as inherently healthier, contrasting it with their perceived stress in urban environments.

It’s far healthier out here than in the city — people breathe better here. (M, 81, rural)

Others described their homes and surroundings as a form of emotional therapy:

I live at the bottom of the Grampian hills. Just looking out at them every morning helps me breathe. (F, 59, urban–rural fringe)

While rural dwellers tended to focus on these advantages, urban participants often emphasised their perceptions of the disadvantages of distance for people living further out.

It must be awful — you’d lose a whole day every time you needed to come in. (F, 68, urban)

Many rural participants accepted long journeys to Aberdeen as an inevitable part of life. Their comments reflected acceptance rather than resentment.

You just plan for it. That’s what you do when you live this far out. (F, 70, rural)

Nonetheless, even those most attached to rural life recognised moments when isolation or travel fatigue took a toll, particularly during treatment.

There were days I didn’t want to drive again — but being home in the quiet made it worthwhile. (F, 59, rural)

For some, temporary relocation to the city or charity accommodation near the hospital was acceptable, but only as a short-term compromise. Most preferred to recover surrounded by familiar people and landscapes.

They offered me to stay in Aberdeen, but I wanted to come home every night — I just wanted to be in my own bed. (F, 58, urban)

Overall, participants portrayed rurality as both burden and buffer. It increased logistical effort but enhanced psychological wellbeing and community belonging. For many, the natural environment, slower pace, and close-knit social ties offset the inconveniences of travel. These reflections underline that rural disadvantage is not experienced purely as hardship but as part of a valued way of life that patients negotiate to maintain dignity, identity, and control during and after treatment.

### Demographic influences

Patterns of experience appear to vary subtly across sex, age, and cancer type, intersecting with geography in complex ways. Men often relied primarily on their spouse or partner for emotional and logistical support. Spouses commonly attended appointments, asked questions, and interpreted medical information, a role participants described with both gratitude and dependence.

My wife came to every visit; I’d have been lost without her asking the right things. (M, 72, rural)

In contrast, women tended to mobilise broader networks that included adult children, friends, or structured peer support such as Maggie’s Centre or local cancer groups. These networks appeared to reduce emotional strain and facilitate social engagement.

My daughters came home to help, and I had friends from Maggie’s checking in. (F, 58, urban)

Cancer type also influenced experiences. Men recovering from prostate surgery highlighted the impact of continence problems on confidence and travel, while colorectal cancer patients described fatigue and digestive urgency that made timing of appointments crucial. Age influenced strategies for coping with travel: older participants emphasised planning such as leaving early to avoid darkness or traffic, while younger or working-age patients focused more on the economic cost of time off and petrol.

These suggested demographic influences, though drawn from a small and heterogeneous sample, underline that gender roles, health status, and life stage can interact with geography to determine how rural disadvantage is experienced.

## Discussion

### Summary of main findings

In this regional qualitative study of patients interviewed after primary treatment and attending oncology follow-up, geography influenced the work of engaging with cancer care after diagnosis, including time, travel, cost, planning, and symptom management during journeys. However, it did not appear to impair the perceived quality of specialist care once accessed. Access and continuity in primary care were variable across settings and influenced trust, help-seeking, and where routine clinical tasks, such as blood tests, were undertaken. Transport infrastructure, local service organisation, and third-sector support, particularly accommodation and transport, appeared to mitigate travel burden in some cases and exacerbate it in others. Rural identity and lifestyle benefits frequently offset perceived disadvantages of distance for rural dwellers.

### Comparison with existing literature

Our findings are consistent with previous UK and Scottish qualitative work showing that patients value accessible, convenient services and that much of the burden falls on patients to organise and coordinate care, regardless of where they live [15, 16]. At the same time, international data suggests that structural barriers, such as care navigation challenges, transport scarcity, and consultation delays, are more pronounced among socioeconomically disadvantaged rural populations, which may explain why these issues were less prominent in our purposive sample which in Scottish terms is likely to be less socioeconomically deprived [2, 17, 18]. Earlier Scottish and Australian studies have also reported that rural patients commonly face longer, more fatiguing journeys, but are willing to trade-off to receive specialist care where it is available [3, 4, 19].

Our data extend the concept of “travel toxicity” by illustrating that distance can compound specific side-effects such as continence problems after prostate surgery and chemotherapy-related fatigue. This has an influence on subsequent appointment timing, willingness to seek help, and decisions about overnight stays [8, 10]. They also complement recent reviews and secondary analyses highlighting rural survivorship challenges and contrasts with urban experiences [7, 9]. Taken together, the literature and our interviews suggest that geography alone does not determine perceived quality of oncology care, but it powerfully influences the work of accessing that care in terms of time, cost, logistics, and symptom management during travel and that these burdens are modulated by deprivation, cancer type, and life stage [5, 11, 15].

Our findings add to emerging international evidence that decentralising elements of cancer care can mitigate “travel toxicity” and enhance patient experience [6]. Within the context of Northeast Scotland, where oncology provision is centralised in a single tertiary centre, these insights highlight the need for regionally adapted service models that bring aspects of surveillance and support closer to home. Policy frameworks should explicitly recognise travel as a modifiable determinant of treatment burden and equity, embedding accessibility and continuity as parallel design priorities in rural cancer care.

### Strengths and limitations

Strengths include a single-centre regional focus enabling contextually grounded implications, use of the Framework Method with investigator triangulation, and concrete service-design signals (e.g. value of AWPR, direct buses, CLAN). Limitations include under-representation of high-deprivation areas and island residents, brief interview durations for some participants, predominance of colorectal cancer, and inclusion of participants post-primary treatment only. Because participants were recruited 6–12 months after diagnosis, and the exact interval from treatment completion to interview was not recorded consistently, the study captures early follow-up and recovery rather than longer-term survivorship or late effects. Most interviews were conducted by telephone, which may have limited observation of non-verbal cues, although this mode may also have facilitated participation for those living further from the research centre. Recruitment was curtailed by the COVID-19 pandemic. Interviews were conducted in 2019–2020, before the widespread adoption of remote and hybrid follow-up models. The findings should therefore be interpreted in that service context.

### Implications for policy and practice

Practical implications arise for service design. Scheduling could be more sensitive to geography: morning appointments and clustering of investigations on the same day would reduce fatigue and unnecessary repeat travel for long-distance patients, while avoiding late-day surgery slots could minimise overnight stays. Localising what can safely be localised, such as pre-chemotherapy blood tests or routine follow-up monitoring within primary care, would further reduce travel burden, provided clear protocols and two-way communication are maintained between local and specialist teams. Transport-aware planning should feature explicitly in health-service policy, ensuring coordination with public transport networks and clear signposting of reimbursement or travel-support schemes. Third-sector organisations, such as CLAN, could be more systematically integrated into care pathways, offering accommodation and transport support as standard components of treatment planning. Digital modalities also have growing potential: telemedicine can substitute for some follow-ups, while virtual peer groups may help counter isolation among remote patients. Finally, support should be tailored to demographic and clinical need—for example, anticipating travel-sensitive side-effects such as incontinence or fatigue and recognising that men and women may access social support differently. Together, these pragmatic steps offer a feasible route to more geographically equitable, person-centred cancer care.

### Future research

Prospective mixed-methods studies should examine interactions between geography, deprivation, sex, and cancer type across the entire pathway and evaluate pragmatic interventions (distance-sensitive scheduling; local hubs for bloods; charity-integrated care pathways) on patient-reported outcomes, utilisation, and equity.

## Conclusions

Rural residence in Northeast Scotland primarily increases the logistical and symptomatic burden of accessing care rather than diminishing specialist care quality. Person-centred scheduling, localised routine care, transport-aware service design, and third-sector partnerships offer practical means to reduce inequity while respecting rural identity and preferences.

## Supplementary Information

Below is the link to the electronic supplementary material.

ESM 1 (DOCX 21.8 KB)

ESM 2 (PDF 116 KB)

ESM 3 (PDF 481 KB)

## Data Availability

Due to the sensitive nature of qualitative interview data, full transcripts are not publicly available. Anonymised extracts may be made available from the corresponding author on reasonable request, subject to ethical approval and appropriate data governance.
