# Supplementary material for: “You lose a day for every appointment”: A qualitative study of how rural versus urban residence shapes cancer care experiences in Northeast Scotland
Source: Support Care Cancer. 2026 Jun 4;34(7):615. doi: 10.1007/s00520-026-10836-2 (PMC13236730; doi:10.1007/s00520-026-10836-2)
Supplement: Supplementary file 2 — (PDF 116 KB) [file 520_2026_10836_MOESM2_ESM.pdf]

Topic Guide

Introduction

Introduce yourself and tell them what you're interested in. This study is about… I'd like to understand your experiences of being diagnosed with cancer and making treatment decisions. I'm particularly interested in whether the place that somebody lives influences any aspects of their experience.

1. **Please tell me about yourself**

   - How old are you?
   - Are you married?
   - Are you working? (If not, what is job status).
   - Who do you live with?
   - Who is your support system made of?

2. **Tell me the story of your diagnosis.**

   - What prompted you to seek help?
   - What influenced your decisions to about how/where/when to seek help?
   - Did anyone else influence that?
   - Do you remember how long you had to wait before you went to your appointments?

3. **Access**

   - What is the approximate distance do you travel to access your respective health facilities? (e.g., GP, cancer centre, etc.)
   - How easy is it, on a normal basis, for you to get medical attention?
   - Consider GP in hours, out of hours, local hospital.
   - Do you think the length of your travel influenced initial consultations – screening access, GP access, investigation access, treatment access.
   - What is your normal form of transportation?
   - Can going to and from these health care facilities be a burden at times?
   - Do you have any barriers/hassles that affect your travel to see the GP?

4. **Tell me about your treatment so far.**

   - How much were you involved in decisions about your treatments?
   - Were there any treatments that you were offered that you decided not to have? (If so, discuss the decision and factors that influenced them).
   - Is/was there anyone supporting you during your treatment?

5. **Did you find any barriers to accepting your course of treatment?**

6. **What has been the most difficult part of treatment for you?**

7. **Were there any practical (logistical) problems (i.e., family and travel) that influenced your treatment decisions?**

8. **Due to your diagnosis, has your lifestyle, relationships or community dynamic changed?**

   - If yes, which parts? Can you elaborate on that?

9. **We're interested in how the place that someone lives affects cancer experiences.**

   - Where you live, do you feel part of a community?
   - Do others in your community speak about cancer?
   - Do others in your wider social network speak about cancer?

- What kind of things are said about cancer in your community?

10. Do you think that urban and rural patients get different care or have different experiences for their cancer treatment?

- How do you think the course of your diagnosis and treatment was/is influenced by where you live?
- Are there any services related to your cancer that concerned you?

11. Policy and Service Organisation:

- What health-related services are important for you to have near?
- Describe the relationship between you and your current GP?
- Do you recall the process your GP followed as soon as they suspected cancer?
